# Fruit and Vegetable Intake and Barriers to Their Consumption among University Students in Kuwait: A Cross-Sectional Survey

**DOI:** 10.1155/2021/9920270

**Published:** 2021-07-09

**Authors:** Dalal Alkazemi, Younis Salmean

**Affiliations:** Department of Food Science and Nutrition, College of Life Sciences, Kuwait University, Alshdadiyah, Kuwait

## Abstract

Data on fruit and vegetable (F/V) consumption and barriers to their intake by Kuwait college students are needed for health promotional activities to curtail obesity and related comorbidities prevalent in Kuwait. This study employed a cross-sectional survey aimed at assessing the median F/V intake in a sample of Kuwait University students to determine its relationship with gender, body weight, college affiliation, and family monthly income and to explore perceived barriers to eating F/V. The median total F/V intake was 2.06, and the median intake of F/V without fries was even lower. Significant gender differences were found in intakes of fruit juice and the percentage of juice from fruit intake, with males consuming more servings per day compared to females. Male students were found to consume proportionately more fried potatoes of total vegetable intake when compared to females, whereas female students were found to consume more vegetables without fries than males. Taste, inconvenience, and lack of knowledge on F/V intake recommendations and preparation methods were among the main barriers to consuming more F/V. College students require encouragement to consume more F/V through targeted campaigns to increase awareness of recommendations, health benefits, and ways to incorporate F/V in their daily diet.

## 1. Introduction

Fruit and vegetable (F/V) consumption is correlated with many health benefits [1, 2]. Diets rich in F/V are protective against lifestyle diseases, such as heart diseases and cancer [3, 4]. Increased F/V consumption is likely to improve the nutritional content of the diet while providing a means to offset the consumption of energy- and fat-dense foods, potentially resulting in lower overall body weight [5].

To prevent coronary heart disease, diabetes, and cancer, most dietary guidelines recommend that adults consume at least five portions (defined as 400 g) of a variety of fruits and vegetables daily [6]. However, few people meet the dietary intake recommendation for F/V [7] partly due to the rapid proliferation of inexpensive fast food that is exacerbated by rising demand for convenience in many countries worldwide [8]. This can lead to an increase in the intake of foods rich in fats, processed carbohydrates, and salt rather than F/V [8, 9].

Young people are more likely to seek convenient food options, lack food preparation skills, and are more prone to consuming less than the recommended amount of F/V and more fast food and energy-dense takeaway meals [10, 11]. These trends can lead to poor nutritional status and obesity-related comorbidities, which are rising public health issues in Kuwait [12, 13]. Therefore, increased intake of F/V is a public health priority requiring significant attention among younger adults. Studies of students and young adults indicate that this age group consumes less than the optimal amount of F/V in various parts of the world [14, 15]. The United States Centers for Disease Control and Prevention (CDC) reported that adults aged 18–24 years consume the fewest vegetables among the national population and that gender differences exist with males typically consuming fewer F/V compared to females [16]. Income status may also influence the intake of F/V [17].

The habit of consuming fewer F/V begins early during childhood and adolescence and leads to decreased F/V intake in adults [18]. Dietary habits are developed and established during young adulthood (ages 18–24 years) when many young people attend college or have significant life changes, including partnering or becoming a parent, causing reduced interest or increased challenges in following a healthy and balanced diet [19]. Therefore, it is a particularly important time to promote healthy eating [19]. The US National College Health Assessment data demonstrates that 94.4% of survey respondents consumed less F/V than recommended [20], consistent with earlier research indicating that 9 out of 10 college students consumed less F/V than recommended [21]. When they are in college, both boys and girls gain independence and self-guidance in their dietary habits. At this age, there is a significant opportunity to develop effective interventions for college students to adopt better nutritional behaviors later in life. However, to develop effective nutrition intervention strategies targeting college students, it is important to understand the current state of F/V consumption among college students in Kuwait, including the impact of demographics, income, and extant barriers to consuming F/V.

Dietary habits of young adults in Kuwait are “unhealthy,” with a higher intake of fat-dense and energy-dense foods and fewer F/V [22]. Among the students from Kuwait University (KU), 26.6% of males and 24.7% of females reported consuming >2 servings a day of whole F/V, respectively, as assessed by a semiquantitative food frequency questionnaire [10]. Dietary screeners are increasingly used to estimate F/V intake as an alternative to more comprehensive dietary assessment methods for population-level community intervention studies and surveillance due to cost and time restraints. The National Cancer Institute's (NCI's) 16-item F/V screener is useful for assessing gross-level estimates, ranking individuals regarding F/V intake, and allows for the median intakes of whole fruits, fruit juice, and vegetables to be assessed separately [23]. This provides better estimates of the contribution of fruits versus vegetables to the overall F/V intake. To date, there is no available quick tool to assess the median F/V intake, and there are no available descriptive data on the details of the types of F/V consumed by college students. Improved understanding of the daily level of F/V intake of young Kuwaitis and their patterns of consumption is required to fill the gap in the extent of knowledge needed for future health promotion efforts targeting college students. Thus, this study aimed to (1) validate the use of a short F/V intake screener among college students using multiple 24-hour dietary recalls (24-hour recalls) as the reference instrument; (2) assess the F/V consumption among male and female students at KU; (3) explore the relationship between body weight and the F/V consumption among college students; and (4) assess potential barriers to eating F/V among Kuwaiti college students.

## 2. Materials and Methods

### 2.1. Study Design and Recruitment

The sampling frame included 34,704 college students enrolled at KU during Spring semester 2016 and aged ≥18 years old. Students <18 years of age and participants who provided incomplete questionnaires were excluded. The study was a cross-sectional survey that comprised a convenient sample of 338 participants (response rate 96.57%); however, only 300 questionnaires were completed (88.76%). The sample consisted of 151 males and 149 females from the various 16 KU colleges. Data were collected from February to April 2016. Participation was voluntary. Recruitment was carried out by research assistants and students. The research team invited participants to participate from the campuses of the 16 KU colleges. The sampling from college campuses was performed using a random cluster sampling method. During recruitment, the purpose of the study and the format of the questionnaire were explained in person and were written clearly as a preface on every questionnaire. Students from each of the 16 university colleges gave their verbal informed consent to participate in the study.

### 2.2. Ethical Approval

Before the start of the study, ethical approval was obtained from the KU Campuses Administration Office, which provided access to all campuses for participant recruitment. The College of Life Sciences Research Committee's ethical approval was obtained, and no further institutional ethics approval was necessary due to the noninvasive nature of the study. This study posed no risk to participants. To ensure data confidentiality, no personal identifiers of participants were collected. The study was conducted according to the ethical standards set by the Declaration of Helsinki. Informed verbal consent to participate was obtained from students who agreed to participate in this study.

### 2.3. Fruit and Vegetable Screener

The 16-item fruit and vegetable screener (FVS) was a modified version of the NCI-FVS evaluated in the Eating at America's Table Study [23]. The final 16-item FVS consisted of frequency and portion size questions that asked about consumption over the past month (i.e., fruit juice, fruit, lettuce/salad, fried potatoes, other potatoes, dried beans, other vegetables, and tomato sauce). Under other vegetables, a shortened version of the 1-cup equivalent visual was provided to aid with portion sizes. Ten frequency category choices ranged from never to 5+ times per day. There were four portion size categories for each food ranging from less than <1/4 cup to >2 cups as well as small, medium, and large portions where applicable (e.g., for fried potatoes, small, medium, and large orders were placed in parentheses next to cup size). The adequate F/V intake was defined as 400–500 g per day or 5 servings of fruit and/or vegetables (80 g each) [6].

### 2.4. Validity and Reliability Study

The FVS was pretested using 24-hour recall administered over 3 nonconsecutive days as the reference method. The three 24-hour recalls were conducted by a dietitian using the automated multiple-pass method and subsequently processed and coded by the investigators. The first recall was taken on the same day as the FVS for a subset of the participants who were not included in the main sample of the study. The remaining recalls were taken through telephone interviews with participants who consented to be contacted for this purpose (*n* = 50). A booklet with household measuring guides to facilitate portion size estimation was provided. We aimed to obtain two 24-hour recalls for weekdays and one for weekends as well as a total of two FVS over 2-3 weeks. Only 32 students (64%) provided the complete required number of recalls and FVS, with no missing information. The ESHA Food Processor software version (11.2) was used for the dietary data entry and analysis, after adding information for local Kuwaiti composite dishes to the software database. The Wilcoxon signed-rank test was used to compare the median of FVS with the average median of the 3 × 24-hour recalls. Deattenuated Pearson correlations were reported for validity, and interclass coefficient was used for reliability.

### 2.5. Barriers to F/V Intake

We obtained perceptions of and attitudes towards F/V, purchase- and preference-related information, and self-reported and socioeconomic information. The survey questions were reviewed for face validity by two faculty members in nutrition and were translated to Arabic and back-translated to English by two different professional translators. Student researchers also reviewed the Arabic version to ensure that the wording was appropriate to Kuwait and understood clearly. Inconsistencies between the two versions were discussed and revised by faculty members. The final questionnaire was piloted among 26 undergraduate students and five women (nonstudents). The content was further revised, accordingly. The final version consisted of 56 items.

Body mass index (BMI) was calculated using the self-reported height and weight. Based on standard adult criteria, BMI was divided into four categories (underweight (<18.5 kg/m^2^), normal weight (18.5–24.9 kg/m^2^), overweight (25–29.9 kg/m^2^), and obese (BMI ≥ 30 kg/m^2^)) as described previously [24].

### 2.6. Statistical Analyses

Normality was evaluated by the Kolmogorov–Smirnov test, which indicated that no variables measured were normally distributed. The participants' characteristics, including demographics, and individual responses to statements were analyzed using descriptive analysis. All variables were expressed as percentages (%) of the study population. Categorical variables were compared using Pearson's chi-square test (*χ*^2^). Fisher's exact tests were used when appropriate, that is, whenever 20% of the expected cell frequencies were ≤5 parametric. For the 16-item FVS, the values for fruits, vegetables, FVS, vegetables without fried potatoes, and F/V without fried potatoes were computed using the scoring system outlined by NCI. Outliers were identified using the variable “Fruit and Vegetable Intake in Cups,” and any observation that was >3 times the interquartile range (IQR; above the 75th percentile or below the 25th percentile) was excluded. Median intake was calculated after the exclusion of participants with outliers. Independent sample median tests were used to test the likelihood that the median values between genders were equal, which were drawn from the same population. Although the median test is regarded as a less powerful alternative to the Kruskal–Wallis analysis of variance test, it is more robust in cases where the dataset contains extreme outliers, as in our study. All reported *p* values were two-sided tests and compared to a significance level of 5%; differences were considered statistically significant at *p* < 0.05. Statistics were performed using SPSS version 25.

## 3. Results and Discussion

### 3.1. Validity and Reliability of the FVS

Of the students who comprised the validity sample (*n* = 32), 52% were female, aged 20–35 years, with 73% from nonscience affiliated colleges and 65% who reported a family income of ≥1000 Kuwaiti dinar (KD) per month (data not shown). Similar findings were observed for the sample of the main study (Table 1). The median values for the total intake of daily F/V for the FVS compared to the 24-hour recalls were 2.3 versus 1.5 for males and 2.1 versus 2.0 for females. The deattenuated Pearson's correlation coefficient for FVS compared with 24-hour estimates for F/V was positive and moderate in strength; *r* = 0.431 for the total validity sample, *r* = 0.532 for males, and *r* = 0.385 for females. The interclass coefficient for test-retest reliability (*n* = 32) for F/V was 0.567 for the total sample and 0.632 and 0.554 for males and females, respectively.

### 3.2. Median Intake of F/V

For the main sample of the study (*N* = 300), the median total F/V intake was 2.06, with the 25th percentile of intake at 1.158 and the 75th percentile at 3.496 (Table 2). Outlier observations comprised approximately 49.66% of the sample (*n* = 149) with 77 (25.7%) students scoring ≤25th percentile (*M* = 0.732, min = 0 and max = 1.16) and 75 students scoring ≥75th percentile (*M* = 5.59, Min = 3.51 and max = 31.77). For F/V (with fries), 15.7% (*n* = 47) of the entire study sample met the 5/day recommendation; however, upon including the variable F/V (without fries), only 13% (*n* = 39) met this recommendation. None of the students met this recommendation after excluding the outliers.

After excluding the outliers, the median total F/V intake did not change for the total sample (2.06 with IQR (1.62–2.53)), with no between-gender differences (Table 3). The median intake for F/V without fries was even lower (1.76 (1.37–2.30)). Statistically significant between-gender differences were observed in intakes of fruit juice, with males consuming more servings per day than females (0.35 (0.11–0.73) versus 0.11 (0.03–0.50), *p*=0.007) and as a percentage of juice from fruit intake (80.69 (66.25–93.72) versus 51.18 (16.59–81.12), *p*=0.002). In addition, male students consumed a higher percentage of fried potatoes of total vegetable intake than females (17.74 (5.29–32.84) versus 8.12 (1.98–23.33), *p*=0.011). Female students consumed more vegetables without fries compared to males (1.35 (0.91–1.94) versus 1.01 (0.64–1.50), *p*=0.040).

**Figure 1:**
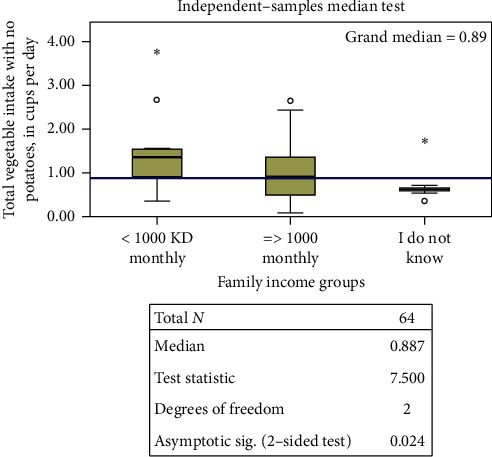
Median test for total vegetable intake with no potatoes according to income categories among males. More than 20% of the cells have expected values less than five.

**Figure 2:**
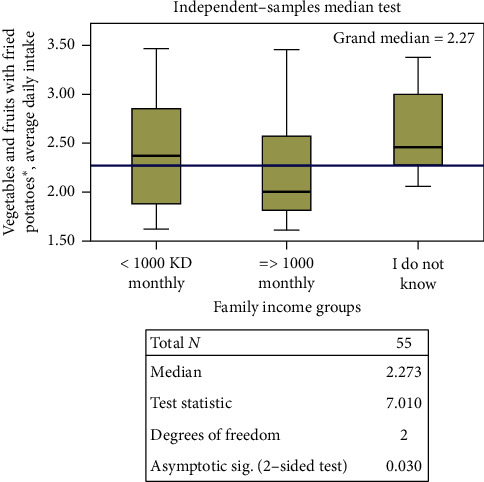
Median test for total vegetable intake with fried potatoes according to income categories among females. More than 20% of the cells have expected values less than five.

Regarding weight according to gender, there were no differences in intakes observed in any of the calculated categories between individuals who were overweight and obese versus those who were not. No differences were observed between college affiliation and health- versus nonhealth-related data (not shown). However, there were statistically significant differences regarding socioeconomic status. Males who reported monthly income >1000 KD had a higher median intake of net vegetables without potatoes compared to those with higher income or those who reported unsure status (Figure 1). Among females, those with lower income and with unsure status had a higher median total F/V intake, with and without potatoes, compared to those with higher monthly income (Figure 2).

### 3.3. Barriers to Consuming F/V

In terms of knowledge regarding F/V intake, students were aware of the association between F/V intake and the reduced risk of obesity (75.5%), diabetes (54.3%), hypertension (49.7%), heart disease (39.1%), depression (28.7%), cancer (28.5%), eye disease (28.5%), Alzheimer's disease (21.3%), premature deaths (17.2%), and arthritis (14.7%). Of the sample, 6.3% reported no knowledge of the association between F/V and risk reduction of any kind. Overall, males were more likely to report no knowledge of this association (9.3% versus 3.4%, *p*=0.03). Knowledge of F/V components that provide health benefits was as follows: 94.0% believed it was vitamins, 66.3% fiber, 45.7% antioxidants, 40.7% minerals, 44% proteins, 63.7% water, 32.3% carbohydrates, 33.7% phytonutrients, and 1.0% none of the listed components. Nevertheless, only 35.7% of the students considered eating F/V a part of a balanced diet, 21.7% consumed them to lose weight, and 22% thought F/V prevents weight gain. More males than females thought they provide energy (30.9% versus 25%, *p*=0.014).

Only 29% of the students reported that they liked the taste of F/V. Students admitted that they were often enticed by other foods instead, even if F/V were available (76.6% males and 80.3% females), and that they forget to buy F/V (45.5% males and 47.9% females). More students found vegetables unappetizing (37.7% males and 35.2% females) compared to those who found fruits unappetizing (27.7% males and 18.3% females). However, students reported that they consumed F/V to stay healthy (77.9% males and 77.5% females) and to prevent diseases (40.3% males and 36.6% females). Females were more likely to eat F/V for weight loss/control (22.5% females and 19.5% males, *p*=0.032) and to get more energy (19.5% males and 35.2% females, *p*=0.014). When asked about the “5 a day” recommendations, only 14.7% reported awareness. More students knew about the “healthy plate” recommendation, with 49% reporting that 50% of the plate should be made up of F/V. Similarly, 42.3% of the students reported that they would typically consume F/V constituting 50% of their plate.

Most of the students considered F/V inexpensive (70.1% males and 83.1% females). Students found that their families had different F/V likes and dislikes (72.7% males and 73.2% females). More than half of the students thought that F/V would go bad before they had consumed them (53.2% males and 64.8% females). Several of them said that they needed more ideas for ways to prepare F/V as part of their meals or as snacks (74.0% males and 74.6% females) and that F/V were time-consuming to prepare (46.4% males and 40.9% females). More females considered precut and prewashed fresh F/V as unhealthy (19.9% males; 30.2% females (*X*^2^ = 4.27, *p*=0.046)); however, most students considered the following unhealthy: fruit cups in syrup (67.5% males; 69.0% females), canned F/V (85.7% males; 84.5% females), and frozen F/V (53.2% males; 59.2% females). Students found that quality F/V were not available at the local stores (40.3% males; 46.5% females) and that there was not a good range of F/V available in cafeterias and restaurants (72.7% males; 56.3% females (*X*^2^ = 6.72, *p*=0.012)). No other statistically significant between-gender differences were observed, except where noted.

## 4. Discussion

To the best of our knowledge, this study is the first to examine F/V intake among a sample of Kuwaiti college students using a validated tool against a reference method and multiple 24-hour recalls. The findings demonstrate that most college adults in Kuwait do not consume F/V as frequently as recommended by the WHO or in sufficient quantities to satisfy other relevant guidelines [6]. Our results indicate that the median total intake of F/V was 2.06 servings per day, with no gender differences when the total F/V intake was assessed.

Our study used the FVS, which displayed a moderate validity and reliability for estimating F/V intake compared with multiple 24-hour recalls, consistent with previous cross-sectional studies [25, 26]. In a multiethnic sample recruited from a behavioral intervention study, when FVS was compared with serum carotenoids as a biomarker of F/V intake and multiple 24-hour recalls, it was observed that the FVS overestimated intake on average by 1.67 servings for men and 2.11 servings for women [27]. The overestimation of intake by the FVS relative to 24-hour recall was associated with a high negative predictive value indicating that the instrument would be effective for identifying subjects with a low intake [27]. Nevertheless, if the instrument exacerbates tendencies to overestimate, then the level of F/V intake among college students in Kuwait is even lower than our estimate, which is more concerning and requires immediate efforts to increase F/V intake through the development of effective interventions and health promotional programs for the prevention of obesity and related comorbidities.

Comparison between studies is problematic due to differing methods of assessment of F/V intake and how the intake is reported, for example, grams per day or frequency of intake [19]. Additionally, most studies assessing F/V intake do not investigate vegetable consumption independent of fruit consumption. Many studies used only the term “vegetables” to refer to the category, whereas other studies divided vegetables into “cooked” and “raw/salads,” and a few were specific, dividing them, for instance, into “green,” “yellow,” “salad,” and “other vegetables” [19]. Moreover, potatoes were sometimes included in the category and sometimes not considered at all. Because of the usability of the NCI-FVS, we were able to calculate the various categories of fruits independently of vegetables and measure the median intake for each category.

Previous reports on Kuwaiti adults estimated the national mean frequency of F/V intake at 3.04 times per day for 2006–2008 [28]. They used a 7-item questionnaire based on the CDC Behavioral Risk Factor Surveillance System, which assessed only the frequency of intake (times per day) without including serving sizes (participants were not told what constitutes a portion/serving), as we did in this study. Hence, the results of our data are not directly comparable to those of the previous study, and their data may misrepresent the level of intake if just based on the frequency per day. In addition, the national surveillance data is based on a sample of older adults, with a mean age of 38.9 years (standard deviation 12.2 years). Further, they identified that the younger age groups fell in the lowest F/V intake [28]; therefore, the Kuwaiti college-aged students would be at a higher risk of not adhering to the dietary guidelines. Indeed, when outliers were not removed, only 15.7% met the recommended level of F/V servings per day when including French fries as a serving of vegetables, whereas 13% met the recommendation of 5 servings per day if the fries were excluded. These findings suggest that most students did not reach the minimum servings of F/V recommended (5 servings F/V a day) [14]. This is a much lower level of adherence than that previously reported among Saudi university students, whereby 22% adhered to 5 or more servings of F/V per day [29].

We observed that when French fries were removed, the median F/V intake of our sample dropped to <2 servings per day. For the total vegetable intake, including fried, boiled, or baked potatoes, the median intake was also low for the total population and per gender equally, with a median intake of 1.61 servings per day. Relative to fruits, vegetables were consumed more; however, most servings of vegetables came from fried, boiled, or baked potatoes, as evident by the significantly decreased servings of vegetables per day when both potatoes and French fries were removed from total intake (−45.96%). Significantly more males reported consuming a higher proportion of servings of French fries per day than females (17.74% versus 8.82%, *p*=0.04). This finding can be explained by a recent study, which demonstrated that most college students in Kuwait reported French fries as their preferred choice of fast food (77.2% women versus 79.6% men), and significantly more men ordered the large size (38.9% versus 4.9%, resp., *p* < 0.001), whereas more women than men reported ordering the regular size (48.8% versus 15.9%, resp., *p* < 0.001) [13].

Servings of leafy green vegetables and other vegetables added to salads, such as carrots and cucumbers, and casseroles or tomato-based stews, such as okra or eggplant, comprised some of the selections consumed by the students. Low consumption of beans was also noted in our sample, with a median <1 serving of beans a day. The low intake of fiber-rich sources was also noted in a recent study, in which most college students reported unhealthy eating behaviors, including low intake of F/V, whole grains, and legumes and a high intake of fast foods, snacks, sweets, and soft drinks [10]. These findings among college students are consistent with those from previous surveys, which indicated that more than 70% or more of Kuwaiti adults consumed below the recommended amount of vegetables, fruits, and legumes, among other rich sources of fiber [28, 30], such as the Eastern Mediterranean Approach for Control of Non Communicable Diseases study [31] and data produced by the Kuwait Nutrition Surveillance System [32].

Our results demonstrated a significantly higher intake of vegetable servings per day (without fries) among female students than among male students (1.35 (0.91–1.94) versus 1.01 (0.64–1.50), *p*=0.018). This finding is consistent with previous results, in which male college students consumed fewer servings of F/V daily than female students (4.3 versus 4.8, *p* < 0.05) [33]. Previous studies have revealed that females eat healthier diets than males. For example, they eat less frequently at fast-food restaurants and consume more servings of fruit and vegetables daily than males [33, 34]. Many factors are associated with a higher intake of vegetables, including normal weight, living in the family home, greater perception of happiness, less pressure and stress, importance given for healthy eating, eating breakfast, lower BMI and blood pressure, higher level of education, more openness to new experiences, early midpoint of sleep, nutrition knowledge, being more active, and lower energy diet density [19]. Among men in the US, the relatively lower F/V intake compared to women was explained by weaker beliefs in the importance of F/V for health as well as decreased confidence in the ability to eat F/V when working, when tired, when watching television, and when other junk foods are available [35].

We also observed that significantly more female students consumed F/V to lose or control their weight (22.5% females versus 19.5% males, *p*=0.032). In the European Prospective Investigation into Cancer Nutrition, a prospective study of 6.5 years including 89,432 men and women from five countries, a significant modest inverse correlation between F/V intake and weight loss was found, such that for every 100 g intake of F/V weight change was −14 g/year [36]. Weight control can motivate increased consumption of F/V as promoted by popular diets and eating plans [37]. Fruits and nonstarchy vegetables are very low in energy since they contain high amounts of water and fiber and can contribute to increased satiety to maintain normal weight [38]. Previous cross-sectional studies have revealed a significant relationship between BMI and vegetable intake, whereby overweight participants had a lower intake of vegetables [39–42]. In this sample, 49% of participants had overweight and obesity; however, no statistically significant differences in the F/V intake levels or the subcategories were observed compared to those who were not overweight or obese because all students consumed low numbers of servings per day. Therefore, the variance in F/V intake was too low to detect differences. Additionally, future studies should include vegetable preparation methods and added ingredients, which may be effect modifiers in this relationship, such as adding more fats, starches, and pastry shells, that is, “samosas” and “dolmas,” which may increase the caloric density of vegetables.

Total fruit intake was less than one serving per day (total median = 0.40 (0.12–1.00)), with 75% of the fruit intake comprising fruit juice, indicating that juices are the most consumed fruit choice with very few whole fruits consumed by both genders. We also observed that males consumed higher servings of fruit juice per day compared to females (81% versus 51%, *p*=0.002). This gender difference in fruit consumption was noted earlier among a US population, where more men consumed ≥2 servings per day than women (36.4% versus 28.7%). Failure to consume whole fruit may be due to issues with practicalities, inconvenience, and the effort required [43]. Fruit juice is a source of sugar lacking fiber, yet juice provides nutrients such as vitamin C, carotenoids, and polyphenols that offer health-related benefits [43]. Encouraging 100% fruit juice consumption to meet recommendations of 5 servings a day may, however, discourage further whole fruit consumption among college students.

This survey identified the current gaps in knowledge on F/V intake among college students. Even though college students in Kuwait seem to be aware of the general health benefits of F/V for well-being and disease prevention, they lacked clarity regarding the specific diet-related diseases, especially cancer. More effort is required to educate students on healthy F/V components and their functions in disease prevention. In addition, we observed that most of the students were not aware of the 5 servings/day F/V recommendation, which may have contributed to the failure to meet their daily recommendation [44]. Among US college students, those who consumed more than the recommended amount of fruit had greater food knowledge than those who reported eating less than the recommended amount [44]. Among Saudi students, greater knowledge of F/V intake and recommendations was observed among those who reported consuming ≥5 servings/day. There is a large gap between the recommended and actual intake of F/V with the current “5 A day” message, and many adults worldwide fall short on the quantity and variety of F/V [45]. However, our results revealed that college students in Kuwait were more aware of the “USDA my plate” recommendation of consuming F/V as 50% of the plate portion and are actively trying to apply this advice. This recommendation, which is “meal-focused,” using the plate as a visual aid, may not be effective for reaching this goal, especially in students' busy lifestyle context, where meals are often skipped. With the average number of main meals less than 2 meals per day [10], the “My Plate” recommendation may not allow students to reach the recommended servings of F/V for the whole day. Healthy eating messages that are more tailored to the lifestyles of college students are key for the successful adoption of healthy eating behaviors. This is important, as they are exposed to a food environment characterized by foods high in fats and sugar, which are energy-dense, on a regular basis.

Many of the barriers to F/V intake identified in our study were related to the tight time-schedules of college students, which hinder their creativity in the kitchen and affect their ability with preparations methods of F/V to avoid quick spoilage, improve palatability, and increase the availability of a quick-grab healthy food when in campus or at home. The perception that F/V is time-consuming to prepare is a frequently cited barrier. Additionally, not having healthy and appetizing F/V options on menu items at the local eateries and college cafeterias was also identified as a perceived barrier to eating more F/V. Improved quality of the on-campus cafeteria food could be achieved by incorporating more dishes with F/V. Habitual consumption of F/V can only be achieved with conscious decisions to choose F/V over other food options. However, students also mentioned that taste was an issue and that they would be more enticed by options other than F/V even if some F/V options existed. Similarly, among college students in the US, 86% of the students indicated that color, taste, and smell were important factors in their food choice [46]. Among college students in the US, family consumption of fruits was highly predictive of the individual's consumption of fruits [47]. For each unit increase in the reported consumption of fruits (vegetables) with the family, the respondents' fruit (vegetable) consumption at school increased by 0.65 (0.30) units compared to the base consumption level of 1.95 (1.82) times of fruit intake per day [47].

Among the factors that influence diet, cost is the main barrier to eating healthy [48]. Various studies have indicated that the relatively high price of F/V is a barrier to healthy eating for people with low incomes. Contrary to these findings, we observed that students, both male and female, with reported lower income were more likely to consume more servings of F/V with or without French fries. Lower-income students may reflect students whose families included more F/V in their dietary patterns. Previous surveys revealed that one of the most important factors in determining someone's F/V intake was whether the habit of eating abundant F/V began in childhood [48]. Another possibility is that the socioeconomic status may be a proxy for culture, and those with lower socioeconomic status maintain a food culture that adheres to traditional foods, thus influencing the level of F/V intake [47]. Data from the Kuwaiti National Nutrition Surveillance revealed that income did not affect nutrient intake [28], which the authors suggest may be due to low food prices and the high availability of locally subsidized foods. The interactions between family meal-related behaviors and income require further exploration in Kuwait to explain these observations.

Behavioral strategies are needed to motivate and enable students to improve their eating behaviors to include more F/V. The college environmental context and conditions affect what and how students eat and what food choices are available on campus. Many intervention approaches may improve college students' dietary habits, including those conducted using in-person, online, or environmental/point-of-purchase messages [49]. For example, in the Netherlands, providing free fruit and vegetables to students at their university was beneficial for those with low habitual intakes [50]. Ha and Caine-Bish [51] used a nutrition course as an intervention for promoting F/V intake among students at Midwestern university aged 18–24 years, whereby the intervention focused on nutrition knowledge related to the prevention of chronic diseases, healthful dietary choices increasing F/V consumption, dietary feedback, and interactive hands-on activities. The researchers observed a significant increase in total fruit and vegetable intake (*p* < 0.005) and a decrease in the intake of French fries (*p* < 0.05). Deliens et al. [52] examined a 10 and 20% meal price increase when choosing French fries and a 10 and 20% meal price reduction when choosing fruit for dessert on university students' purchasing behavior in an on-campus restaurant. Researchers found that there was an absolute reduction by 10.9 and 21.8%, respectively, in the purchase of French fries, while there were absolute increases in fruit purchases of respectively 25.1 and 42.4% (all *p* < 0.001). For Kuwaiti college students, interventions may need to address the other important determinants of students' food choices, such as taste and desire, knowledge regarding health recommendations, and product accessibility and availability. The likelihood of intervention success may increase with offering healthy and tasty alternatives to French fries and offering a variety of fresh and appealing fruits, improving the food quality away from home.

These findings are concerning, especially given that the newer guidelines recommend a much higher level of F/V consumption at 7–13 servings (3.5–6.5 cups) daily for adults, depending on gender and activity level [14]. Consuming higher amounts of F/V decreases the risk of being overweight [53], heart disease [54], and certain types of cancers [55]. Intake of F/V is also associated with the prevention of type 2 diabetes, stroke, hypertension, cataracts, chronic obstructive pulmonary disease, and diverticulosis [56].

The generalizability of our results to all college students in Kuwait may be limited, as we did not include private universities and colleges. In addition, our sample may be biased by those interested in participating in the study versus students who were busier and less accessible. All data were based on participants' self-report and, thus, subject to recall bias.

This study is timely, providing updated information on the F/V intake level of college students in Kuwait. This information may help determine efficient and age-appropriate strategies for health promotional activities to increase F/V intake to prevent obesity and related chronic diseases [57]. Typically, public health policies have focused on education, hence aimed at increasing knowledge [46]. Increased communication regarding the importance of family traditions, like eating at the dinner table, may have a greater impact than increasing information on the number of servings of F/V people should consume [46].

## 5. Conclusions

Understanding the barriers to students' F/V intake can help decision-makers improve food choices available in on-campus eateries. This study is the first to provide a detailed breakdown of what constitutes F/V intake among college students, which can further assist in tailoring nutrition education interventions targeting specific subgroups of F/V, including green salads, fruit juice, mixed dishes, and different processing methods for preparation or preservation and their impact on students' health outcomes, such as weight, cognitive function, stress management, mood, and sleep.

## Figures and Tables

**Table 1:** Demographic characteristics of participants (*N* = 300**)**.

Age groups (years)	<20	60 (20)
	20–35	229 (76.3)
	>35	11(3.7)

Gender	Male	151 (50.3)
	Female	149 (49.7)

Weight status (BMI)	Not overweight or obese	153 (51)
	Overweight and obese	147 (49)

Marriage status	Single	251 (83.7)
	Married	49 (16.3)

Family income (KD)	<1000 KD monthly	55 (18.3)
	≥1000 KD monthly	177 (59)
	Not sure	68 (22.7)

Colleges	Law	9 (3)
	Arts	38 (12.7)
	Sciences^*∗*^	40 (13.3)
	Medicine, public, and allied health^*∗*^	26 (8.7)
	Engineering and petroleum	33 (11)
	Education	71 (23.7)
	Sharia and Islamic studies	20 (6.7)
	Business administration	3 (1)
	Pharmacy^*∗*^	1 (0.3)
	Dentistry^*∗*^	3 (1)
	Social sciences	4 (1.3)
	Life sciences^*∗*^	39 (13)
	Architecture	1 (0.3)
	Computer science and engineering	12 (4)

^*∗*^Science-related colleges. KD: Kuwaiti dinar, BMI: body mass index in kg/m^2^.

**Table 2:** Median and interquartile ranges of intake of fruits and vegetables (cups/day) among the total population (*N* = 300) and according to gender.

Daily intake (categories)	Total (*n* = 300)	Males (*n* = 151)	Females (*n* = 149)	*P* values^*∗*^
Fruit juice	0.21 (0.07–0.50)	0.21 (0.07–1.00)	0.11 (0.03–0.50)	0.002
Fruit units	0.05 (0.017–0.25)	0.05 (0.02–0.25)	0.05 (0.00–0.25)	0.733
Fruit cups	0.00 (0.00–0.00)	0.00 (0.00–0.00)	0.00 (0.00–0.00)	0.977
Leafy greens/salad	0.20 (0.05–0.39)	0.13 (0.05–0.25)	0.21 (0.03–0.50)	0.566
French-fried potatoes	0.16 (0.04–0.39)	0.25 (0.05–0.50)	0.11 (0.03–0.39)	0.133
Potatoes (baked, boiled, or mashed)	0.16 (0.05–0.59)	0.16 (0.05–0.59)	0.16 (0.03–0.59)	0.986
Cooked dried beans	0.05 (0.02–0.19)	0.05 (0.02–0.21)	0.05 (0.01–0.16)	0.118
Other vegetables	0.16 (0.05–0.50)	0.16 (0.05–0.38)	0.16 (0.05–0.75)	0.894
Tomato sauce	0.11 (0.02–0.25)	0.11 (0.02–0.25)	0.11 (0.02–0.29)	0.916
Vegetable soup	0.07 (0.00–0.21)	0.07 (0.02–0.24)	0.06 (0.00–0.21)	0.135
Total vegs and fruits (with fries)^*∗*^	2.08 (1.16–3.51)	2.06 (1.16–3.28)	2.06 (1.11–3.69)	1.00
Vegs and fruits (without fries)	1.76 (0.97–2.97)	1.74 (1.01–2.76)	1.79 (0.88–2.99)	1.00
Total veg (all listed)	1.61 (0.80–2.97)	1.49 (0.76–2.93)	1.67 (0.84–3.08)	0.678
Vegs (without the beans)	1.44 (0.75–2.73)	1.49 (0.75–2.93)	1.67 (0.76–2.98)	0.248
Vegs (without fries)	1.22 (0.60–2.21)	1.00 (0.60–2.21)	1.35 (0.59–2.36)	0.065
Vegs (without potatoes)	0.85 (0.37–1.57)	0.77 (0.39–1.50)	0.97 (0.32–1.59)	0.356
% potato fries of the total veg	14.27 (3.85–29.80)	16.22 (4.92–33.28)	8.82 (1.99–23.54)	0.011
Total fruit	0.41 (0.17–1.01)	0.50 (0.17–1.21)	0.32 (0.10–0.75)	0.204
Total fruit (no juice)	0.11 (0.02–0.25)	0.11 (0.02–0.25)	0.11 (0.02–0.25)	0.918
% juice of fruit	67.63 (34.45–92.61)	79.70 (45.10–92.66)	64.17 (21.86–90.33)	0.076

^*∗*^Independent samples Mann–Whitney *U* test, *p* < 0.05. Veg: vegetables.

**Table 3:** Median and interquartile ranges of fruit and vegetable intake (cups/day)^a^.

Daily intake (categories)	Total (*n* = 151)	Males (*n* = 80)	Females (*n* = 71)	*P* values^*∗*^
Fruit juice	0.21 (0.07–0.50)	0.35 (0.11–0.73)	0.11 (0.03–0.50)	0.007
Fruit units	0.05 (0.02–0.25)	0.05 (0.02–0.25)	0.11 (0.02–0.25)	0.179
Fruit cups	0.00 (0.00–0.00)	0.00 (0.00–0.00)	0.00 (0.00–0.00)	0.789
Leafy greens/salad	0.21 (0.05–0.39)	0.20 (0.05–0.25)	0.25 (0.05–0.50)	0.167
French-fried potatoes	0.20 (0.04–0.39)	0.25 (0–0.40)	0.11 (0.03–0.38)	0.274
Potatoes (baked, boiled, or mashed)	0.16 (0.05–0.50)	0.16 (0.05–0.57)	0.16 (0.05–0.50)	0.983
Cooked dried beans	0.05 (0.02–0.20)	0.05 (0.02–0.21)	0.05 (0.02–0.16)	0.130
Other vegetables	0.16 (0.05–0.38)	0.16 (0.05–0.38)	0.16 (0.05–0.59)	0.892
Tomato sauce	0.13 (0.03–0.25)	0.13 (0.03–0.25)	0.13 (0.03–0.25)	0.983
Vegetable soup	0.06 (0.00–0.21)	0.07 (0.00–0.21)	0.06 (0.00–0.11)	0.442
Veg and fruits (with fries)^*∗*^	2.06 (1.62–2.53)	2.05 (1.63–2.54)	2.06 (1.62–2.53)	0.939
Veg and fruits (without fries)	1.76 (1.37–2.30)	1.75 (1.29–2.20)	1.80 (1.41–2.36)	0.687
Total veg (all listed)	1.61 (1.20–2.15)	1.53 (1.09–2.07)	1.67 (1.67–2.24)	0.315
Veg (without the beans)	1.43 (1.07–1.96)	1.37 (0.95–1.88)	1.52 (1.21–2.00)	0.291
Veg (without fries)	1.24 (0.79–1.66)	1.01 (0.64–1.50)	1.35 (0.91–1.94)	0.018
Veg (without potatoes)	0.87 (0.46–1.36)	0.80 (0.45–1.32)	1.09 (0.38–1.44)	0.088
% potato fries of the total veg	14.70 (4.38–29.02)	17.74 (5.29–32.84)	8.12 (1.98–23.33)	0.040
Total fruit	0.43 (0.17–0.92)	0.50 (0.20–1.06)	0.34 (0.10–0.71)	0.565
Total fruit (no juice)	0.08 (0.02–0.25)	0.05 (0.02–0.25)	0.11 (0.02–0.25)	0.167
% juice of fruit intake	75.05 (29.58–90.50)	80.69 (66.25–93.72)	51.18 (16.59–81.12)	0.002

^*∗*^Independent samples Mann–Whitney *U* test, *p* < 0.05. ^a^Total population with outliers removed, based on the variable total vegetables and fruits (with fries) (*N* = 151) and according to gender. Veg: vegetables.

## Data Availability

The datasets used and/or analyzed during the current study are available from the corresponding author upon reasonable request.
